# Application of Supervised SOM Algorithms in Predicting the Hepatotoxic Potential of Drugs

**DOI:** 10.3390/ijms22094443

**Published:** 2021-04-24

**Authors:** Viktor Drgan, Benjamin Bajželj

**Affiliations:** 1 Laboratory for Cheminformatics, Theory Department, National Institute of Chemistry, Hajdrihova 19, 1001 Ljubljana, Slovenia; benjamin.bajzelj@ki.si; 2 Biotechnical Faculty, University of Ljubljana, Jamnikarjeva 101, 1000 Ljubljana, Slovenia

**Keywords:** classification, hepatotoxicity, QSAR, supervised neural network

## Abstract

The hepatotoxic potential of drugs is one of the main reasons why a number of drugs never reach the market or have to be withdrawn from the market. Therefore, the evaluation of the hepatotoxic potential of drugs is an important part of the drug development process. The aim of this work was to evaluate the relative abilities of different supervised self-organizing algorithms in classifying the hepatotoxic potential of drugs. Two modifications of standard counter-propagation training algorithms were proposed to achieve good separation of clusters on the self-organizing map. A series of optimizations were performed using genetic algorithm to select models developed with counter-propagation neural networks, X-Y fused networks, and the two newly proposed algorithms. The cluster separations achieved by the different algorithms were evaluated using a simple measure presented in this paper. Both proposed algorithms showed a better formation of clusters compared to the standard counter-propagation algorithm. The X-Y fused neural network confirmed its high ability to form well-separated clusters. Nevertheless, one of the proposed algorithms came close to its clustering results, which also resulted in a similar number of selected models.

## 1. Introduction

Quantitative structure–activity relationship (QSAR) modelling is based on the similarity principle that structurally similar compounds have similar physicochemical properties. Therefore, compounds with similar structures can be expected to have similar effects in biological systems. QSAR methods are important complements to in vitro and animal testing methods. In the drug development process, they may provide a quick and cost-effective assessment of the compound properties. Although the QSAR methods cannot completely replace all in vitro and animal testing methods, they present an important contribution to the reduction in animal tests. Therefore, QSAR methods have also been recognized as important for the risk assessment of chemicals. In addition to directly predicting the property of compounds using a QSAR model, the read-across method can be used to predict the same endpoint based on the known endpoint value of a structurally similar compound or group of similar compounds. Self-organizing maps (SOMs), also known as Kohonen neural networks, are known for their ability to group objects according to their similarity and can be used to project objects from multidimensional to two-dimensional space [1]. Supervised Kohonen neural networks are an extension of SOMs that have an additional (output) layer of neurons that is trained to predict an endpoint. Probably the simplest extension of SOMs are counter-propagation neural networks (CPANNs), where the Kohonen layer of neurons is used to determine the position of the winning neuron, and the output layer is used to predict the endpoint. In CPANN, the endpoint is not used to determine the winning neuron or to correct the neuron weights in Kohonen layer, but only to correct the weights in the output layer. One can occasionally obtain models that are difficult to interpret because no relationship between the independent variables and the endpoint is apparent when comparing the model weights in the Kohonen and output layers, which is especially difficult when endpoint clusters in the output layer are not well formed. During the training process, SOMs can form clusters of objects that preserve topological relationships when projections of objects are made from multidimensional to lower dimensional space. The data can be grouped into the correct cluster, but clusters are often scattered on the map leading to overlapping clusters [2]. Therefore, new learning algorithms have been developed to improve the predictive ability and interpretation of supervised SOM models.

The behavior of supervised Kohonen networks in overdetermined datasets was studied by Xiao et al. [3]. Their observation confirmed the superior behavior of supervised SOM over supervised *k*-means clustering, which are closely related. SOM is practically a *k*-means clustering algorithm when the neighborhood function (kernel) of SOM becomes zero [3,4]. The better performance of SOM models over *k*-means clustering apparently arises from the neighborhood information that is lost when the neighborhood becomes zero.

In the work of Melssen et al. [5], examples of clustering results using different learning algorithms for SOM models are given. To obtain a desirable response surface of the model, they proposed an X-Y fused network and a bi-directional Kohonen neural network. Compared to the checkerboard response obtained for some of the examples shown in their paper with the counter-propagation and supervised Kohonen neural network, the proposed algorithms produced a response surface with well-formed class clusters. In the X-Y fused network, the endpoint property was used to determine the winning neuron and weight the learning rate based on the similarity of the object to the neuron in the Kohonen and output layers. In the bi-directional Kohonen neural network, the corrections of weights in both layers are not made all at once, as in X-Y fused networks, but sequentially, with two passes of objects through the network. In the first pass, the winning neuron is determined based on the similarity in the output layer and the weights in the Kohonen layer are updated using all the objects. This is followed by the second pass, where the winning neuron is determined based on the similarity in the Kohonen layer and then the weights in the output layer are corrected for all objects.

Recently, Torres-Alegre et al. [6] proposed a concept of metaplasticity in SOMs (AMSOMs) for modification of the learning process using Gaussian function implementing the metaplasticity concept. Previously, they introduced the concept to improve the backpropagation algorithm [7] in the training of multilayer perceptron artificial neural networks. The idea was to give higher relevance to infrequent patterns and reduce in cases of the frequent ones. Performance evaluation showed that the standard SOM method performed slightly better than AMSOM when using smaller networks, while AMSOM performance showed better results when using larger networks. The observed learning progress was slower in AMSOM, with larger variabilities observed during training, however better performances were obtained at larger network sizes.

The above-mentioned authors tried to improve learning strategies of SOM with different approaches. One of the important tasks in QSAR is finding appropriate chemical space representation. Approaches for utilizing information on infrequent patterns, for example, can boost the model, but without adequate chemical representation one may have difficulties building a good model due to so-called activity cliffs. The activity cliffs were generally defined as pairs of structurally similar active compounds with a large difference in potency [8]. They represent steep changes in the structure−activity relationship (SAR), so they hinder QSAR modeling [9], although on the other hand they can identify small chemical modifications that determine activity of compounds [10] and are thus very important.

The aim of this work was to develop a learning strategy for counter-propagation artificial neural networks that improves the training capabilities of the network and leads to the good formation of clusters on the SOM top-map. In the training and testing phase, the determination of the winning neuron is performed in the same way as in the standard CPANN model, independently of the endpoint. Different learning strategies were used and genetic algorithm optimization of CPANNs was performed to evaluate the relative learning strengths of the algorithms. Weight correction algorithms of the standard CPANN are proposed, where the difference between scaled object variable and the corresponding scaled model weight is used to adjust the amount of weight correction. Initially, the weight corrections resemble classical CPANN algorithm, and the scaling gradually gains importance in weight correction during the training process. The proposed algorithms may reduce the effect of structural outliers on the training. They were used for the classification of drugs from LiverTox database and showed improved clustering abilities compared to standard CPANN.

## 2. Results and Discussion

Genetic optimizations of neural network models were performed using hepatotoxicity datasets with 268 and 49 initial descriptors in the training set and four neural network training algorithms described in Section 3.2. *Theoretical Background*. The neural networks used were the standard counter-propagation neural network (CPANN), the X-Y fused neural network, and two proposed learning algorithms called CPANN-v1 and CPANN-v2. The same initial conditions and the same model selection criteria were used for all optimizations with the same initial number of descriptors. The same number of optimizations were performed using all four learning algorithms. Results obtained for individual optimizations are available in the file the “optimization_results.zip”. Sensitivity, specificity, and clustering formation score (CFS) values are given in supplementary file separately for each of the training algorithms used. The following tables, Table 1 and Table 2, show the number of selected models that were obtained when different training algorithms and optimization criteria were used in the optimization process.

From Table 1, it can be seen that with the proposed CPANN-v2 algorithm, the largest number of selected models was found overall. The number is slightly larger than the number of selected models found with the X-Y fused neural network. With the standard CPANN model, the smallest number of the model was found, and the CPANN-v1 algorithm resulted somewhere in the middle between the largest and lowest numbers of the selected models found. Significantly lower numbers are observed in Table 2 than in Table 1, which was to be expected because the large reduction in the number of initial descriptors in the training set reduced the amount of valuable information available to build a model. In this case, using the CPANN-v1 algorithm resulted in the largest number of selected models found. Again, the use of the standard CPANN algorithm resulted in the smallest number of models found, while the use of the X-Y fused network and CPANN-v2 algorithm resulted in approximately the same number of models found. The only difference between the CPANN-v1 and CPANN-v2 algorithm is larger emphasis of the endpoint on the weight correction in the CPANN-v2 algorithm given by a factor and considering differences between the scaled object endpoint variable and corresponding scaled response weight in the weight correction equation.

The comparison of the number of selected models in Table 1 and Table 2 shows that the largest number of selected models was found using the optimization criterion OC2. The same number of selected models was found using optimization criteria OC1 and OC2 when using 49 descriptors, as shown in Table 2. The optimization criterion OC2 was also the most complex optimization criterion used. Nevertheless, optimization criterion OC4 resulted in the lowest number of selected models, indicating that trying to minimize the differences between minimal and maximal sensitivity and/or specificity may not result in better models. The optimization criterion OC4 was derived from a simpler optimization criterion OC3, but fewer models were found by OC4 than by OC3.

**Figure 1:**
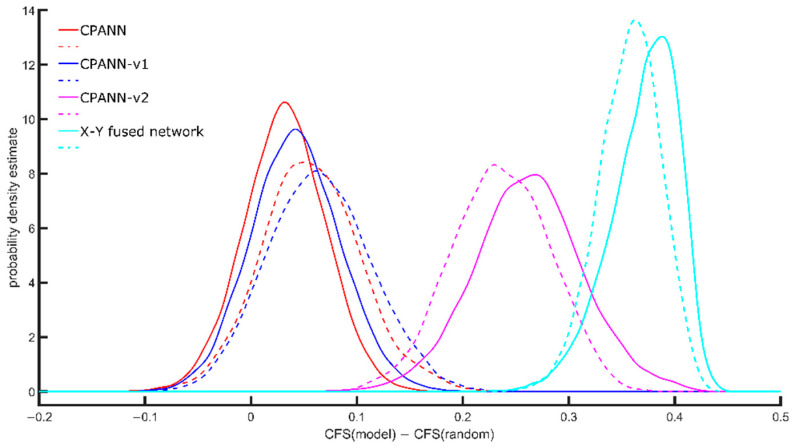
Probability density estimate of differences CFS(model)–CFS(random) obtained for models developed using different training algorithms. Solid lines represent the distribution for selected models obtained from optimizations with 268 descriptors in the training set, dashed lines represent distributions for selected models obtained from optimizations with 49 descriptors in the training set.

The modifications to the standard weight correction equations were made in the CPANN-v1 and CPANN-v2 training algorithms to develop models with better cluster formations than when the standard CPANN algorithm was used. With the better formation of clusters, the interpretation of the models may be simpler. X-Y fused neural networks are known to generate such models. However, during training, the endpoint variables (targets) are used along with independent variables (descriptors) to select the winning neuron. The activation of a neuron during training depends significantly on the endpoint variable, which is removed when predictions are made with an existing model. In the proposed CPANN-v1 and CPANN-v2 algorithms, the winning neurons are selected independently of the endpoint variables during training and when making predictions, in the same way as when using standard CPANNs. The models developed using standard CPANN, X-Y fused neural network, CPANN-v1 and CPANN-v2 were evaluated using the clustering formation score (CFS) described in Section 3.4. *Evaluation of Cluster Formation of Models* to compare their relative ability to form clusters. The results of the evaluation are shown in Figure 1. The CFS depends on the size of the network and the number of neurons giving response to a specific class; therefore, the CFS of a model (CFS(model)) was compared with the average CFS(random) that was calculated for random distribution of the same responses on the network with the same size. The calculation of the average CFS(random) was performed using 100 random distributions of the response values. Figure 1 shows the probability density estimate obtained for the differences between CFS(model) and CFS(random). The solid lines indicate the distributions obtained using selected models developed during the optimizations with a set of 268 descriptors, and the dashed lines indicate the distributions obtained using the selected models developed during the optimizations with a set of 49 descriptors.

The X-Y fused network shows the best ability to form clusters. The proposed CPANN-v2 algorithm is the next one with good ability for the formation of clusters. CPANN-v1 algorithm shows slightly better ability than the standard CPANN algorithm. When using 49 descriptors during the optimizations, the formation of clusters improved with standard CPANN and CPANN-v1 algorithm compared to results obtained when 268 descriptors were used during optimizations. A small decrease in the formation of clusters was observed for the CPANN-v2 algorithm and X-Y fused network models.

The selected models differed in the size of the network and in the descriptors that were present in each of the models. Among these models, the most frequently selected descriptors in optimizations were identified. For optimizations performed with different training algorithms, the 10 most frequently selected descriptors were identified separately. Then, four lists of the most frequent descriptors were compared, and some common descriptors were identified. This was conducted separately for optimizations performed with 268 and 49 descriptors. The common descriptors that were found are listed in Table 3. These descriptors can be considered as the most important descriptors for predicting hepatotoxic potential of drugs.

**Figure 2:**
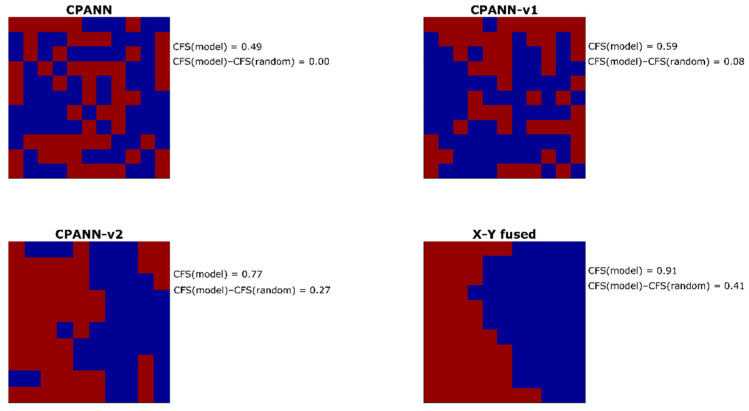
Response surfaces of selected models obtained from optimizations with initial 268 descriptors in sets. Red color indicates hepatotoxic prediction and blue color indicates non-hepatotoxic prediction of the models.

From the entire pool of the selected models, one model was selected for each of the algorithms. The models with high and comparable prediction performances were selected among the models obtained from optimizations with 268 initial descriptors in the sets. For the selection, average sensitivity and specificity values were considered that were calculated from 100 models built neural network training parameters and different permutations of objects in the training set. The average sensitivity values for the external validation set were 0.80 for CPANN model, 0.89 for CPANN-v1 model, 0.89 for CPANN-v2 model, and 0.81 for the X-Y fused model. Average specificity values for the external validation set were 0.82 for CPANN model, 0.84 for CPANN-v1 model, 0.85 for CPANN-v2 model, and 0.87 for the X-Y fused model. The response surfaces (predicted classes for each neuron) of these models are shown in Figure 2. Level plots for the models are available in the supplementary file “level_plots.zip” and the top-mapsthe in the supplementary file “top-maps.zip”. Model weights and predictions of the models are available in the supplementary file “model_weights_and_predictions.xlsx”. Each square on the response surface corresponds to response of one neuron. Red color indicates the neurons where the model predicts hepatotoxic class, and the blue color indicates non-hepatotoxic prediction. On the right side of each response surface, calculated clustering formation score values of the models (CFS(model)) and the differences CFS(model)–CFS(random) are given. Higher values of the differences CFS(model)–CFS(random) are expected for the models, resulting in better separation of classes. According to the values of the differences CFS(model)–CFS(random), the selected models can be sorted in the following order (from the highest to the lowest value): X-Y fused, CPANN-v2, CPANN-v1 and CPANN. It is visible from Figure 2 that a better separation of hepatotoxic and non-hepatotoxic classes is obtained with the X-Y fused and CPANN-v2 networks than with CPANN or CPANN-v1 networks.

Misclassified external set compounds from each of the four models were inspected. The results are shown in Table 4. Half of the misclassified cases were misclassified once. In Table 4, the second column shows identification numbers of compounds from the training that excited the same neuron as the misclassified external set compound. There are nine cases where at least two compounds from the training set excited the same neuron as the external set compounds and have different hepatotoxic activity. Two such cases are found in predictions for the model built using the CPANN-v2 algorithm, one case in the model built with the X-Y fused network algorithm, and the remaining six cases are attributed to the other two algorithms.

Additional sets were used to further evaluate the results obtained by different training algorithms. A number of models were built using different sets for classification of compounds into a class with high or low affinity to the target proteins. The models were built for angiotensin-converting enzyme (ACE), acetylcholinesterase (ACHE), benzodiazepine receptor (BZR), cyclooxygenase-2 (COX2), dihydrofolate reductase (DHFR), glycogen phosphorylase b (GPB), thermolysin (THER), and thrombin (THR). Table 5 shows the number of selected models obtained for the additional sets that were selected when using three different performance thresholds (0.70, 0.75 and 0.80). In Table 5, the numbers in bold indicate the largest number of selected models for a protein target at a selected threshold value. From Table 5, it can be seen that X-Y fused and CPANN-v2 network models most frequently achieved the largest number of selected models.

**Figure 3:**
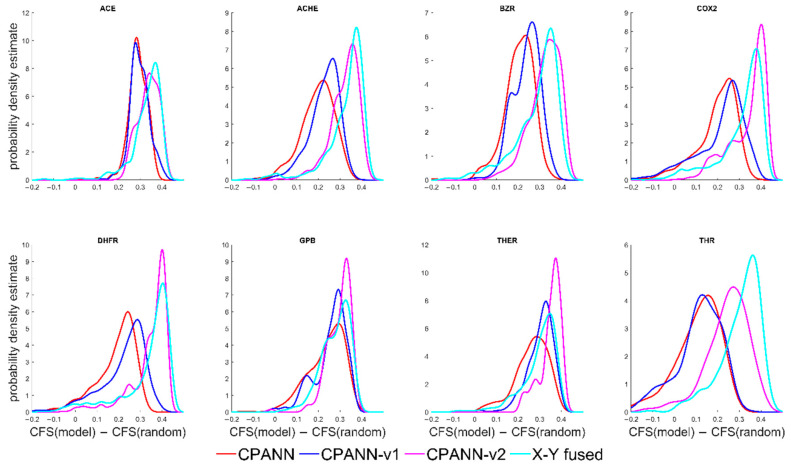
Probability density estimates of differences CFS(model)–CFS(random) obtained for models developed using the additional sets.

Clustering formation scores and the differences CFS(model)−CFS(random) were calculated for the models. The probability density estimates of the differences CFS(model)−CFS(random) are shown in Figure 3. In the Supplementary Material, supplementary file “results_for_additional_sets.zip” contains files with information about the performances and CFS values for the models that were built for the additional sets. In Figure 3, the position of peaks in the distributions of CFS(model)−CFS(random) for the CPANN-v2 and X-Y fused networks are shifted to higher values than for CPANN and CPANN-v1, which is similar to the results shown in Figure 1. However, there are smaller differences in the distributions and larger overlaps of the peaks are obtained for these models.

## 3. Materials and Methods

### 3.1. Datasets

The information about the potential of drugs for causing liver injury was obtained from the LiverTox database [11]. The structures of the compounds used to calculate descriptors were collected from the PubChem database [12] and were manually curated. If the structures contained ions, the counter ions were removed, and a neutral form of the compound was used. The drugs with hepatotoxicity likelihood scores A, B, C, D and E were used for the mapping of compounds according to their structural similarity with Kohonen neural network. The mapping was performed based on 0–2D molecular descriptors calculated with Dragon 7 software [13]. Detailed descriptions of the descriptors implemented in Dragon are given in the literature [14]. From the entire set of drugs, only the compounds with molecular weight up to 850 g/mol were used and any compounds containing metals or elements B, Br, I or P were removed. Only the descriptors calculated for all the selected compounds and with no more than 70% of equal values were used. The initial dataset with 433 compounds and 268 descriptors is available in the Supplementary Materials as Supplementary_File_1.

Based on the Kohonen top-map with 8 × 8 neurons, and using 268 molecular descriptors, an external validation set of compounds was selected. The top-map showing the initial distribution of LiverTox classes is given in the Supplementary Materials as supplementary file “initial_distribution_of_livertox_classes.png”. The corresponding top-map with compound identification numbers is given in supplementary file “initial_distribution_of_compound_IDs.png”. CPANNatNIC software [15] was used for drawing top-maps used for dividing compounds into sets. The top-map presenting the selection of the external validation set compounds is given in the Supplementary Materials as supplementary file “validation_set_selection.png”. On the top-map, the compounds that were selected for the validation set are marked with red color (compounds belonging to classes A, B, and E). At the same time, the compounds marked with orange color (compounds belonging to classes C and D) were removed from the set of compounds. After the selection of the external validation set, the remaining compounds were mapped using SOM to select train and test sets. The corresponding top-map is shown in the supplementary file “internal_set_selection.png”, where red color indicates compounds selected for the test set and orange color indicates compounds from classes C and D that were removed from the set. The test set was randomly split into two sets “test set 1” and “test set 2”, which were used as the internal test set and internal validation set when performing genetic algorithm optimizations of CPANN models. The splitting of the compounds into an internal test set and internal validation set is presented with the top-map presented in supplementary file “internal_test_and_internal_validation_set.png”. On the top-map, the compounds selected for the internal test set are marked with red, and the compounds selected for the internal validation set are marked with orange. The remaining compounds were used for the training set, except the compounds belonging to classes C and D. Based on the likelihood score categories, only the compounds from categories A (“well known” to cause liver injury), B (“highly likely” to cause liver injury) and E (“not believed or unlikely” to cause liver injury) were used for model development. Categories C and D were lacking adequate numbers of reported cases for drugs causing liver injury. All the sets with normalized descriptor values are given in the Supplementary Materials as Supplementary_File_2. Then, a smaller set of descriptors was selected from the previous sets of 268 descriptors by removing descriptors one by one until the maximal pairwise correlation coefficient in the training set was not greater than 0.5. This procedure resulted in 49 descriptors and new sets were created using the same compounds in the sets as before. The new sets with 49 normalized descriptors are available in the Supplementary Materials as Supplementary_File_3.

### 3.2. Theoretical Background

#### 3.2.1. Kohonen Neural Networks

Detailed descriptions of Kohonen neural networks, also known as self-organizing maps (SOMs), can be found in the literature [1,16]. A brief description of the training algorithm is given in this section, because it presents the foundations for the neural network algorithms used in this study and the Kohonen top-map was used for the selection of compounds into the sets mentioned in the previous section.

Kohonen neural networks belong to unsupervised learning methods where the information about the target property is not needed to develop a model. Kohonen neural networks consist of one layer of neurons. Each neuron can be represented as a one column matrix containing model weights that correspond to the independent variables (molecular descriptors) of the data used to train the network. The training of the network entails identification of the winning neuron (also known as the central neuron or the best matching unit) and subsequent correction of the weights in the layer of neurons. The winning neuron is usually determined as the neuron with the shortest Euclidean distance between the independent variables describing the object (molecular descriptors) and the corresponding neuron weights. When the winning neuron is determined, the weights are updated according to Equation (1).

w(*t*, *i*, *j*, *k*) = w(*t* − 1, *i*, *j*, *k*) + η(*t*) ∙ h(*i*, *j*, *t*) ∙ (o(*k*) − w(*t* − 1, *i*, *j*, *k*)) (1)

In Equation (1), the new value of the weight calculated in iteration *t*, w(*t*, *i*, *j*, *k*), corresponding to variable *k* of the object, o(*k*), is calculated by adding a correction to the existing weight value from the previous iteration, w(*t* − 1, *i*, *j*, *k*). At the beginning of training, the weights are initialized with random values, usually in the range (0,1). The position of the neuron is given by the coordinates (*i*, *j*), and *t* represents the iteration step when a single object is used for the correction of weights in the neural network model. On the other hand, one epoch of training means that each object in the training set was used in the training exactly once. Learning rate function, η(*t*), is usually monotonically decreasing. The neighborhood function, h(*i*, *j*, *t*), describes how the correction of the weights is changing during the training with respect to the distance from the winning neuron. Neighborhood function used in this study was triangular, with initially the largest possible neighborhood, which was decreasing in size so that in the last iteration only the weights of the winning neuron were corrected.

#### 3.2.2. Counter-Propagation Neural Networks

The description of counter-propagation artificial neural networks (CPANNs) is given in detail in the article written by Zupan et al. [17]. CPANNs are extensions of Kohonen neural networks with an additional output layer of neurons (also known as the Grossberg layer). In the output layer of neurons, the weights are corrected using Equation (1), the same as in the Kohonen layer, except now the object variables represent endpoint (target) values of the objects. The position of the central neuron in the output layer is obtained by simple projection of the neuron location from the Kohonen layer to the output layer.

**Figure 4:**
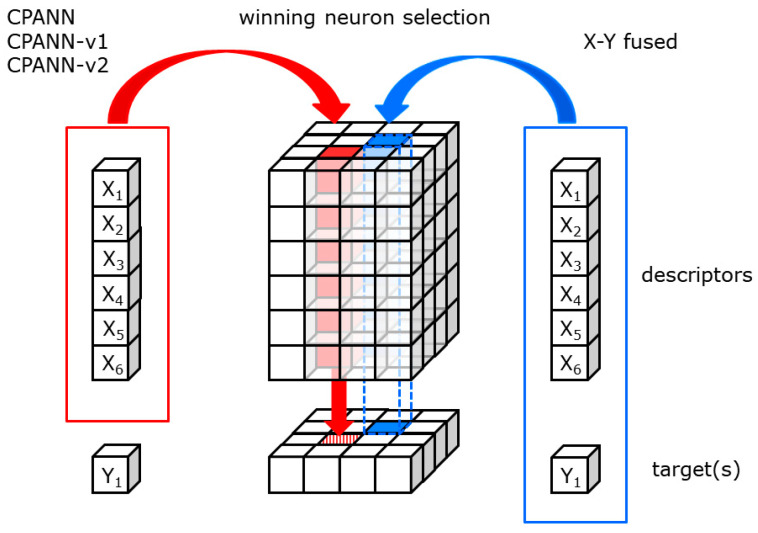
Schematic representation of neural network architecture. The arrows show winning neuron selection during the training process. CPANN, CPANN-v1 and CPANN-v2 use descriptors to determine similarity, while the X-Y fused network also considers target values.

The learning algorithm used was the same as in a previous study [18]. The modification of the standard algorithm was used due to the significantly biased dataset containing a larger number of compounds from non-hepatotoxic class. The training procedure involving random subsampling of the training set compounds was used, which is explained in detail in the article [18]. Random subsampling was applied to all supervised learning algorithms used in this study (CPANNs, X-Y fused networks, and modified CPANNs) to obtain a comparable number of compounds from hepatotoxic and non-hepatotoxic class in each epoch. One epoch derives a slightly different meaning from the one for standard CPANNs, designating the number of training iterations where each object from the random subsample (and not the entire training set as in the standard CPANNs) was used exactly once [18]. A schematic representation of neural network architecture is given in Figure 4. The same representation can also be considered for CPANN-v1, CPANN-v2 and the X-Y fused network described in the following sections. The same procedure is used to obtain the prediction from these networks. An object that is represented by a set of descriptor values is compared with all neurons in the neural network, and the most similar neuron is selected as the central neuron. The position of the neuron is projected on the output layer and the prediction is obtained from the output layer. During the training process, the central neuron is determined in the same way except for the X-Y fused network. During the training of the X-Y fused network, the target variable is also used to determine the central neuron, as schematically indicated in Figure 4 (blue color for X-Y fused network).

#### 3.2.3. X-Y Fused Networks

X-Y fused networks are presented in the paper written by Melssen et al. [5]. In such networks, dependent and independent variables of the training set are used to determine the best matching unit according to Equation (2), and the weights are corrected as in standard Kohonen networks. In Equation (2), S_Fused_(*i*,*k*) represents similarity between input object pair (*X_i_*,*Y_i_*) and unit (neuron) k of Xmap and Ymap, where Xmap represents weights corresponding to the independent variables (as in the Kohonen layer) and Ymap represents weights corresponding to the output variable (as output layer in CPANN). Adaptive learning can be used to improve learning with the weighting factor *F* calculated using Equation (3). The similarities are normalized; therefore, the weighting factor has the largest value, 2, for a perfectly matched object, and the lowest value, 1, for an object with no match. Using adaptive learning, the correction of the weight is increased by a factor of two when a perfect object is presented to the network. During the training, the value of α(*t*) linearly decreases with epoch *t*, so that at the end of the training both maps contribute equally to the determination of the winning neuron.

S_Fused_(*i*,*k*) = α(*t*) ∙ S(*X_i_*,Xmap*_k_*) + (1 − α(*t*)) ∙ S(*Y_i_*,Ymap*_k_*) (2)

F = 2 − (α(*t*) ∙ S(*X_i_*,Xmap*_k_*) + (1 − α(*t*)) ∙ S(*Y_i_*,Ymap*_k_*)) (3)

#### 3.2.4. Modified CPANN Version 1

A modification of the CPANN learning algorithm is presented in this section and will be called CPANN-v1. The algorithm resembles a standard CPANN learning algorithm. The determination of the winning neuron is identical to the determination of the winning neuron in Kohonen neural networks or CPANNs. Modifications of the training algorithm are made to weight corrections. Specifically, Equation (1) is modified to the following Equation (4) by adding multiplication term m(*t*, *i*, *j*, *k*). The value of m(*t*, *i*, *j*, *k*) is calculated using Equation (5).

w(*t*, *i*, *j*, *k*) = w(*t* − 1, *i*, *j*, *k*) + m(*t*, *i*, *j*, *k*) ∙ η(*t*) ∙ h(*i*, *j*, *t*) ∙ (o(*k*) − w(*t* − 1, *i*, *j*, *k*)) (4)

m(*t*, *i*, *j*, *k*) = 1 − (1 − p(*t*)) ∙ ABS[scaled(o(k)) − scaled(w(*i*, *j*, k))] (5)

In Equation (5), ABS indicates the calculation of absolute value of the term in the square brackets, scaled(o(k)) is the range-scaled value of the object variable *k*, scaled(w(*i*, *j*, k)) is the range-scaled value of the object weight corresponding to variable *k*, and p(*t*) is linearly decreasing during the training. In this study, it decreased from 1 towards 0 during the training. The value of scaled(o(k)) is range-scaled based on all values of variable *k* in the training set. The value of scaled(w(*i*, *j*, k)) is the range-scaled weight value based on all values in the level of weights corresponding to the variable *k*. In the special case where all values (variable or weight values) are equal, the scaled value is set to 1. Both range-scaled values, scaled(o(k)), and scaled(w(*i*, *j*, k)), are in range [0,1]; thus, m(*t*, *i*, *j*, *k*) also holds value in range [0,1].

#### 3.2.5. Modified CPANN Version 2

This section presents another modification of the standard CPANN algorithm, which is an extension of the CPANN-v1 algorithm and will be called CPANN-v2. This extension was intendent to give higher importance to the endpoint variable during the training. An additional factor, using the scaled endpoint variable, scaled(o(target)), and corresponding scaled weight, scaled(w(*i*, *j*, target)), was added to Equation (5), and Equation (6) was obtained:

m(*t*, *i*, *j*, *k*) = [1 − (1 − p(*t*)) ∙ ABS[scaled(o(k)) − scaled(w(*i*, *j*, k))]] ∙ [1 − (1 − p(*t*)) ∙ ABS[scaled(o(target)) − scaled(w(*i*, *j*, target))]] (6)

### 3.3. Optimizations of Neural Network Models

Optimizations of neural networks were performed using the genetic algorithm (GA) and four different learning algorithms: standard CPANNs, X-Y fused networks, CPANN-v1, and CPANN-v2. Detailed descriptions of genetic algorithms can be found in the literature [19]. Descriptions of four optimization criteria were used, and all learning algorithms had the same initial parameters set for optimization runs. Optimizations were performed using LiverTox datasets with 268 descriptors and 49 descriptors. Due to the imbalanced dataset, biased towards a larger number of compounds from non-hepatotoxic class, 33% of compounds from the non-hepatotoxic class and 66% of compounds from the hepatotoxic class were used to equalize the number of hepatotoxic and non-hepatotoxic compounds in each subsample. Optimization runs were conducted by using four optimization criteria, denoted as OC1, OC2, OC3 and OC4, which were calculated by means of Equations (7)–(13). Optimization criteria were calculated using training and internal test sets. Factor f(Nsel) was used to consider the number of selected descriptors (Nsel) in the optimization criterion from the total number of descriptors in the training set (Ndes). The value of *a* in Equation (11) was set as 1 and 4 when using the training sets with 49 or 268 descriptors, respectively.

OC1 = (MCC(train) + MCC(test)) ∙ f(Nsel)(7)

OC2 = ABS[MCC(train) ∙ MCC(test)] ∙ (1 − ABS[MCC(train) − MCC(test)]) ∙ f(Nsel)(8)

OC3 = Min_val ∙ f(Nsel)(9)

OC4 = OC3 ∙ (1 − (Max_val − Min_val)) ∙ f(Nsel)(10)

f(Nsel) = 1 − *a*∙(Nsel − 1)/Ndes (11)

Min_val = MIN[sensitivity(train), sensitivity(test), specificity(train), specificity(test)](12)

Max_val = MAX[sensitivity(train), sensitivity(test), specificity(train), specificity(test)](13)

In Equations (7)–(13), MCC denotes the Matthews correlation coefficient calculated for train (MCC(train)) or internal test sets (MCC(test)), ABS denotes the absolute value of the value in the square brackets, MIN denotes the minimal value in square brackets, and MAX denotes the maximal value in square brackets.

**Figure 5:**
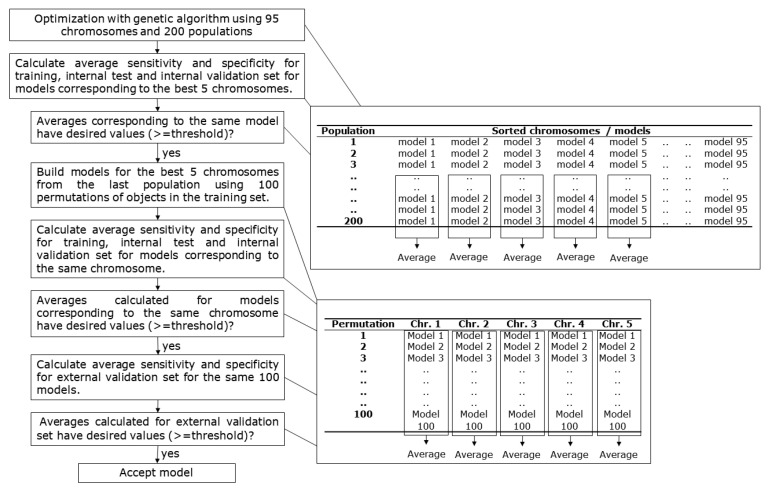
Schematic presentation of the model selection process.

A schematic representation of the model selection process is given in Figure 5. Each GA optimization run lasted for 200 chromosome populations. A total of 95 chromosomes were used in each population, and the best five chromosomes were passed unchanged to the next population of chromosomes. The genetic algorithm was used to select descriptors and the parameters used to train the network (number of training epochs, size of the network, minimal and maximal learning rate). The same initial optimization conditions were applied when performing optimizations of neural networks with different training algorithms. Selection of the models was made using the following criteria. First, the average value of sensitivity and specificity for the train, internal test set, and internal validation set had to be at least 0.7 for one of the best five chromosomes in the last 20 populations (the calculation of averages is presented with the top table on the right side of the scheme in Figure 5). From the optimizations that satisfied the criteria, the best five chromosomes of the last population were taken, and 100 models were built for each chromosome using different permutations of train set compounds during training. Average values of sensitivity and specificity for the train, internal test, and internal validation sets were calculated for 100 models (the calculation of averages is presented in the bottom table on the right-hand side of the scheme in Figure 5). The chromosomes that resulted in minimal average values of 0.7 were further evaluated using the external validation set. The same criterion with a minimal value of 0.7 for sensitivity and specificity was applied to the external validation set. The models from optimization runs that satisfied all the criteria were considered as acceptable.

### 3.4. Evaluation of Cluster Formation of Models

Different algorithms were used to build neural network models. It was expected that due to different rules for the correction of weights, the algorithms had different abilities to develop models with well-formed clusters that can be observed on response surface. To evaluate the extent of cluster formation for a model, here we define a measure which we call *clustering formation score* (CFS). The clustering formation score was calculated using Equation (14).

(14) $$\mathrm{CFS}=1-\frac{\;\sum_{j=1}^{Ny}\sum_{i=1}^{Nx-1}ABS\left[R\left(i,j\right)-R\left(i+1,j\right)\right]+\sum_{i=1}^{Nx}\sum_{j=1}^{Ny-1}ABS\left[R\left(i,j\right)-R\left(i,j+1\right)\right]}{2NxNy-Nx-Ny\;}$$

In Equation (14), *i* and *j* represent the coordinates of a neuron with position (*i*,*j*), and response *R*(*i*,*j*). *Nx* and *Ny* indicate the number of neurons in the *x*- and *y*-directions of a 2D map. The response of the neuron was obtained from model weights *w*, for the weight level corresponding to the endpoint. In the calculations, the actual values of *R*(*i*,*j*) were 0 or 1, where *R*(*i*,*j*) = 1 was taken for the neuron response greater than 0.5, and *R*(*i*,*j*) = 0 was used elsewhere. The equation is applicable to networks with non-toroidal architecture, which were used in this study. The CFS value of 0 corresponds to a response surface with a checkerboard response. The CFS value of 1 corresponds to the response surface of a model where all neurons give the same response, because all the differences under the summation signs in Equation (14) become zero.

### 3.5. Calculations on Additional Datasets

The training algorithms presented in this paper were applied to build classification models on additional datasets. The datasets were obtained from Sutherland’s datasets [20] comprising inhibitors of angiotensin-converting enzyme (ACE), acetylcholinesterase (ACHE), benzodiazepine receptor (BZR), cyclooxygenase-2 (COX2), dihydrofolate reductase (DHFR), glycogen phosphorylase b (GPB), thermolysin (THER), and thrombin (THR). For all the compounds in these datasets, descriptor values were obtained from previous publications [15,21]. The same division of compounds into training and test sets was used as in the previous papers. For classification purposes, the compounds were split into two classes based on the median value of all activity values in training set. The compounds with activity values above the median activity value of all training set compounds were put into the high-activity class, other compounds were put into the low-activity class. Classification models were built using different initial training conditions (number of epochs, network size, minimal and maximal learning rate). The same initial training conditions were used to build the models with all four algorithms presented in this paper.

## 4. Conclusions

In this work, modelling the hepatotoxic potential of drugs was performed using supervised self-organizing neural network algorithms. Two new weight-correction methods were proposed to improve the formation of clusters on the top-map. Achieving good cluster separation can be helpful for the interpretation and understanding of neural network predictions. The results obtained using new algorithms were compared with results obtained using a standard counter-propagation neural network and X-Y fused neural network. Clustering formation score, defined in the paper, was used to assess the relative ability of algorithms to obtain good separation of clusters. The results showed better clustering abilities of the proposed algorithms than the standard counter-propagation neural network, and the CPANN-v2 algorithm was close to the results of the X-Y fused neural network. The number of models found by the proposed CPANN-v2 algorithm was slightly larger than the number of models found by the X-Y fused network, indicating good training capabilities of the algorithm. Similar performance behavior was observed when models were built for additional sets. Considering the separation of classes, smaller differences were observed among the algorithms. Nevertheless, similar trends were observed as with the LiverTox dataset.

## Figures and Tables

**Table 1:** Number of selected models obtained when using an initial set of 268 descriptors.

Algorithm	Optimization Criterion				
	OC1	OC2	OC3	OC4	Total
CPANN	41	121	12	5	179
X-Y fused	71	122	65	84	342
CPANN-v1	76	112	44	38	270
CPANN-v2	77	140	78	61	356

**Table 2:** Number of selected models obtained when using an initial set of 49 descriptors.

Algorithm	Optimization Criterion				
	OC1	OC2	OC3	OC4	Total
CPANN	8	4	16	8	36
X-Y fused	34	22	4	7	67
CPANN-v1	36	19	49	10	114
CPANN-v2	10	43	5	2	60

**Table 3:** List of common descriptors.

Optimization Set	Common Descriptors
268 descriptors	Mi—mean first ionization potential (scaled on Carbon atom) GATS5i—Geary autocorrelation of lag 5 weighted by ionization potential nCS—number of total secondary C(sp3) CATS2D_09_AA—CATS2D Acceptor-Acceptor at lag 09
49 descriptors	NNRS—normalized number of ring systems GATS3m—Geary autocorrelation of lag 3 weighted by mass GATS5m—Geary autocorrelation of lag 5 weighted by mass GATS6m—Geary autocorrelation of lag 6 weighted by mass JGI4—mean topological charge index of order 4 JGI5—mean topological charge index of order 5 F04[N-O]—Frequency of N - O at topological distance 4

**Table 4:** Misclassified external set compounds.

External Set Compounds	Training Set Compounds	Distance ^a^	Neuron Position	Algorithm
Acrivastine 910 (−)	249 (+), 95 (+)	0.43	[5,5]	CPANN
Amoxicillin 885 (+)	804 (−), 348 (−)	0.53	[3,8]	CPANN-v1
Cabazitaxel 812 (−)	855 (+), 738 (+)	1.03	[1,2]	CPANN-v1
Cabazitaxel 812 (−)	51 (+)	0.88	[4,11]	X-Y fused
Clobazam 725 (−)	/	/	[8,10]	CPANN-v1
Didanosine 623 (+)	739 (−), 162 (+)	0.57	[2,7]	CPANN
Didanosine 623 (+)	739 (−), 430 (−)	0.43	[6,9]	X-Y fused
Eliglustat 587 (−)	726 (+)	0.40	[10,3]	CPANN
Enalapril 583 (+)	427 (−)	0.77	[5,7]	CPANN
Enalapril 583 (+)	235 (−)	0.52	[6,5]	CPANN-v2
Ezogabine 540 (−)	249 (+), 95 (+)	0.54	[5,5]	CPANN
Ezogabine 540 (−)	95 (+)	0.46	[4,8]	CPANN-v2
Ezogabine 540 (−)	/	/	[5,4]	X-Y fused
Fentanyl 532 (−)	421 (−), 269 (−), 11 (+)	0.23	[4,5]	CPANN-v1
Iloperidone 451 (−)	594 (+), 237 (−), 24 (+)	0.39	[5,7]	CPANN-v2
Imipramine 438 (+)	890 (+), 257 (−)	0.18	[3,6]	CPANN-v1
Isoniazid 431 (+)	253 (−)	0.64	[9,8]	CPANN
Isoniazid 431 (+)	841 (−), 357 (−), 253 (−)	0.28	[11,7]	X-Y fused
Metaproterenol 351 (−)	/	/	[5,5]	X-Y fused
Naratriptan 328 (−)	588 (−), 146 (−), 24 (+)	0.52	[7,1]	CPANN
Nimodipine 316 (−)	756 (+), 348 (−)	0.42	[4,3]	CPANN-v2
Oxazepam 288 (−)	805 (+), 480 (−)	0.53	[5,10]	CPANN
Oxybate 287 (−)	913 (+), 841 (−), 39 (+)	0.52	[3,11]	CPANN-v1
Riociguat 165 (−)	887 (+), 886 (−), 713 (+)	0.60	[7,1]	CPANN-v1
Tegaserod 90 (−)	756 (+), 39 (+)	0.58	[8,5]	CPANN
Torsemide 36 (−)	756 (+), 39 (+)	0.48	[8,5]	CPANN
Torsemide 36 (−)	95 (+)	0.32	[4,8]	CPANN-v2
Torsemide 36 (−)	319 (+), 152 (+)	0.46	[3,7]	X-Y fused

(+) and (−) indicate actual class of the compounds: (+) hepatotoxic class, (−) non-hepatotoxic class. ^a^ Normalized Euclidean distance (ED) to the nearest compound from training set that excited the same neuron. Normalized ED was calculated as ED/√n, where *n* is the number of descriptors used in the model.

**Table 5:** Number of selected models obtained for the additional sets.

Protein Target	CPANN			CPANN-v1			CPANN-v2			X-Y Fused		
	Threshold ^a^			Threshold ^a^			Threshold ^a^			Threshold ^a^		
	0.7	0.75	0.8	0.7	0.75	0.8	0.7	0.75	0.8	0.7	0.75	0.8
ACE	256	64	11	229	58	9	**293**	**113**	**35**	244	83	16
ACHE	46	6	**1**	30	5	0	33	3	0	**81**	**17**	**1**
BZR	37	**7**	0	**48**	2	0	**48**	**7**	0	48	6	0
COX2	151	29	**1**	131	25	0	14	1	0	**223**	**45**	0
DHFR	330	95	5	371	123	6	**654**	**377**	**65**	256	40	5
GPB	**36**	**21**	**8**	30	9	3	22	8	0	8	0	0
THER	139	38	16	143	**63**	**29**	147	35	13	**208**	53	13
THR	2	2	0	2	1	0	14	3	0	**26**	**9**	0

The following number of models were developed for the protein targets: GPB, THER and THR 300 models, ACE and ACHE 360 models, BZR 420 models, COX2 660 models and DHFR 720 models. ^a^ indicates minimal sensitivity and specificity for training and test sets needed to select a model. The numbers in bold indicate the largest number of selected models for a protein target at a selected threshold value.

## Data Availability

Data is contained within the article or supplementary material.

## Supplementary Materials

The following are available online at https://www.mdpi.com/article/10.3390/ijms22094443/s1, optimization_results.zip: File containing information about sensitivity, specificity and clustering formation score for optimizations; level_plots.zip: File containing level plots of four selected models; top-maps.zip: File containing top-maps of four selected models; model_weights_and_predictions.xlsx: File containing model weights of four selected models and prediction results of the models; results_for_additional_sets.zip: File with the results obtained for additional sets; initial_distribution_of_livertox_classes.png: Top-map showing initial distribution of classes; initial_distribution_of_compound_IDs.png: Top-map showing initial distribution of compounds; validation_set_selection.png: Top-map indicating selected external validation set compounds; internal_set_selection.png: Top-map indicating compounds selected for internal sets; internal_test_and_internal_validation_set.png: Top-map indicating division of compounds into internal test and internal validation set; Supplementary_File_1.xlsx: Initial dataset with all compounds and calculated descriptors; Supplementary_File_2.xlsx: Dataset with 268 descriptors; Supplementary_File_3.xlsx: Dataset with 49 descriptors.
