# Supplementary figures and images for: Application of Supervised SOM Algorithms in Predicting the Hepatotoxic Potential of Drugs
Source: Int J Mol Sci. 2021 Apr 24;22(9):4443. doi: 10.3390/ijms22094443 (PMC8123051; doi:10.3390/ijms22094443)

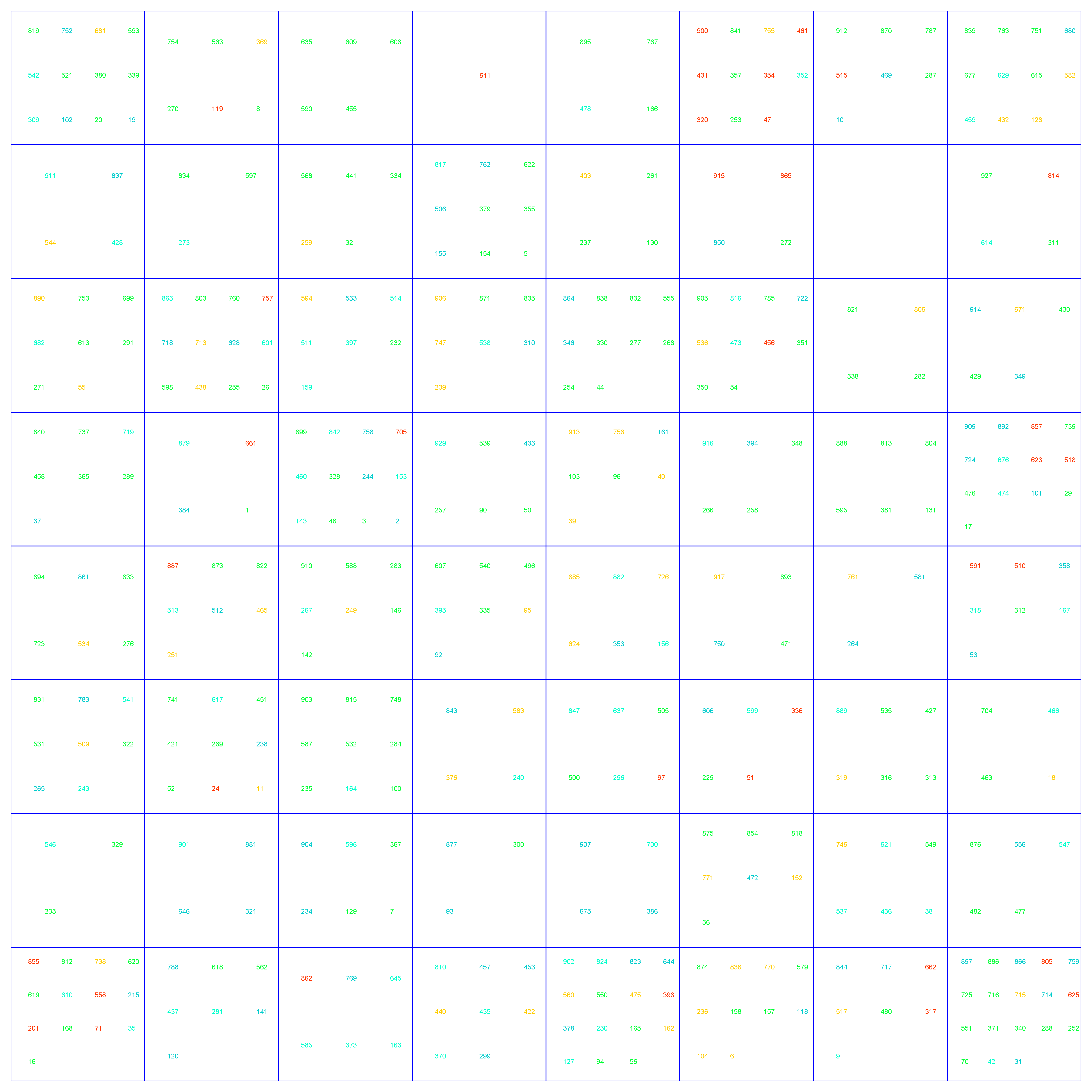

Supplement: Supplementary file 1 [file ijms-22-04443-s001.zip › initial_distribution_of_compound_IDs.png]

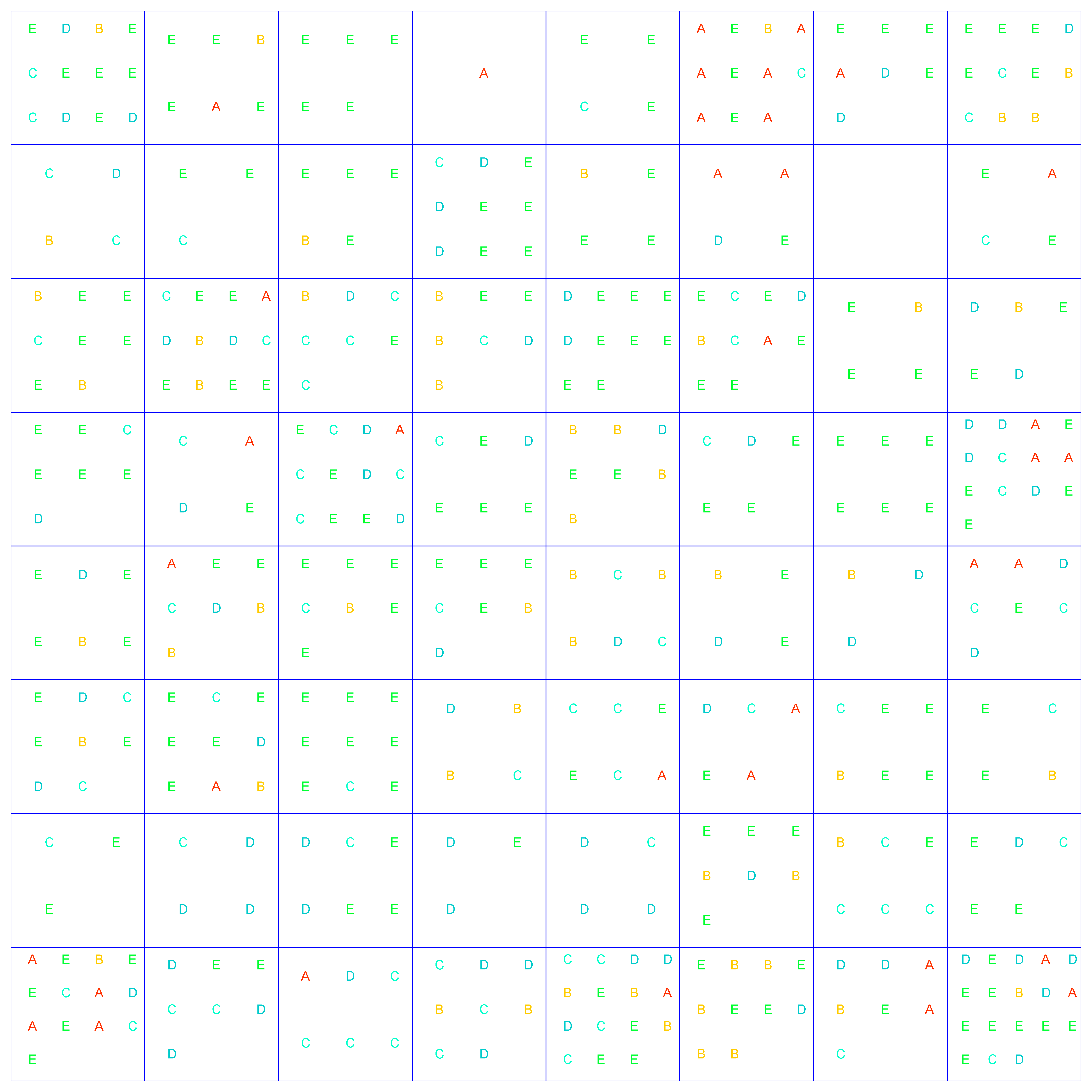

Supplement: Supplementary file 1 [file ijms-22-04443-s001.zip › initial_distribution_of_livertox_classes.png]

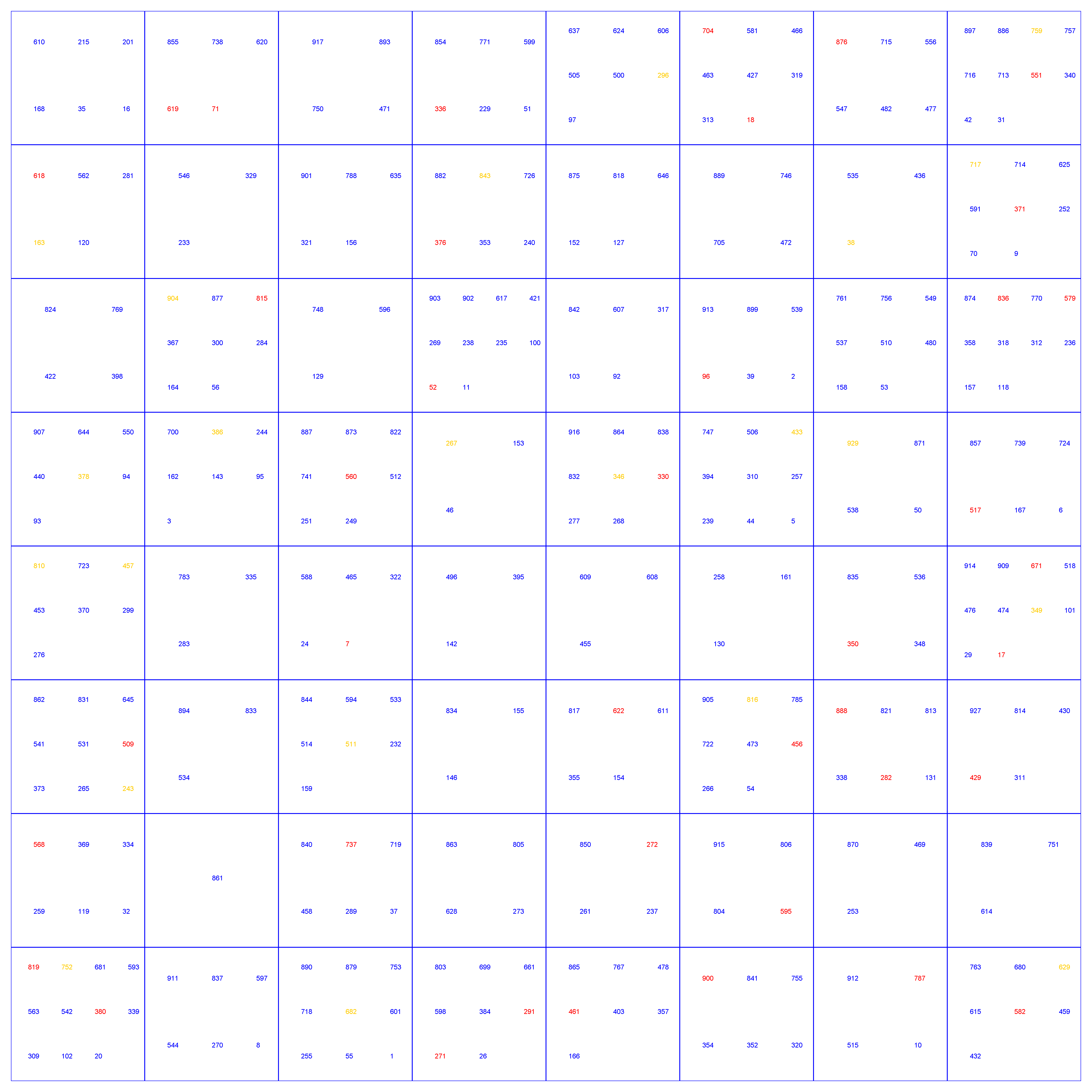

Supplement: Supplementary file 1 [file ijms-22-04443-s001.zip › internal_set_selection.png]

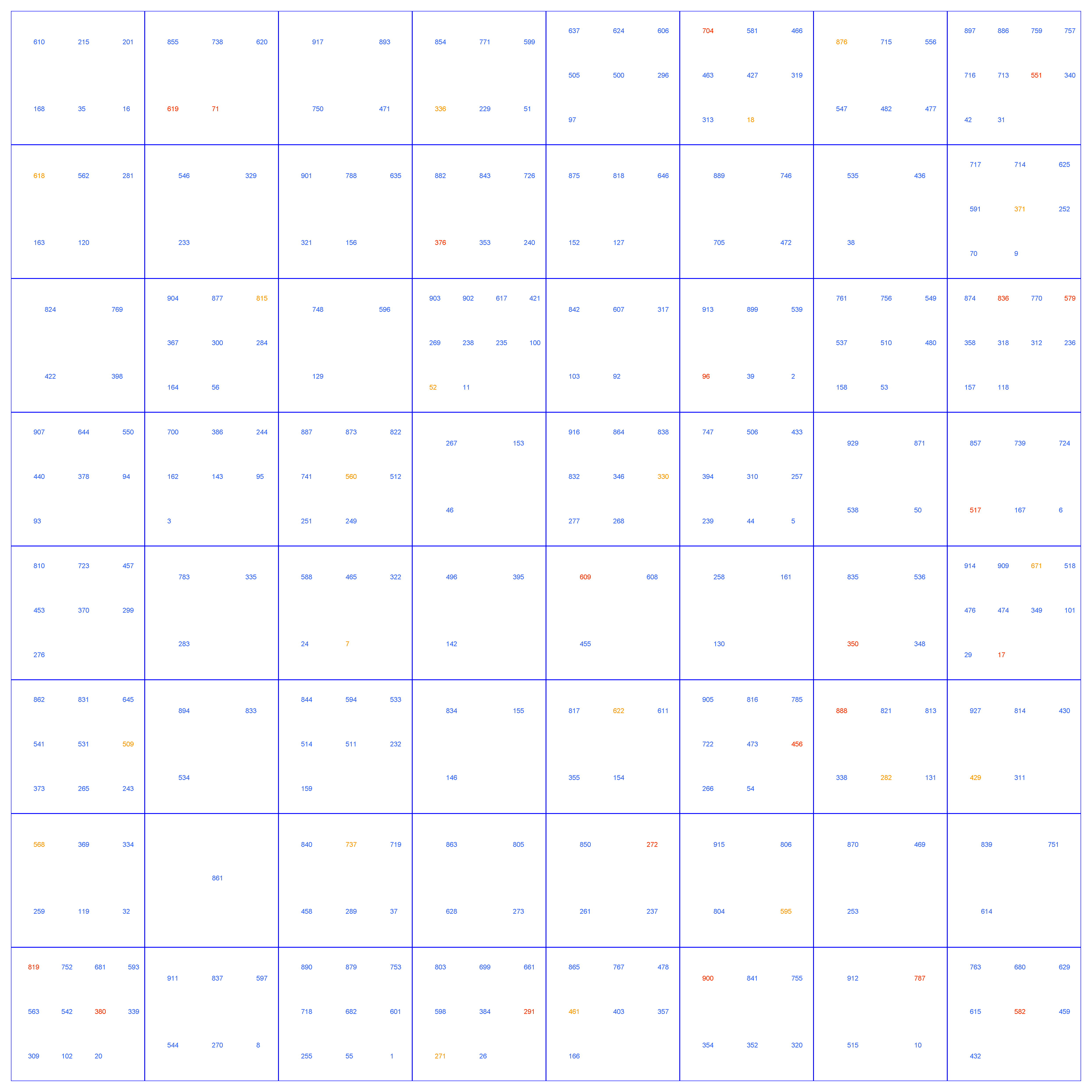

Supplement: Supplementary file 1 [file ijms-22-04443-s001.zip › internal_test_and_internal_validation_set.png]

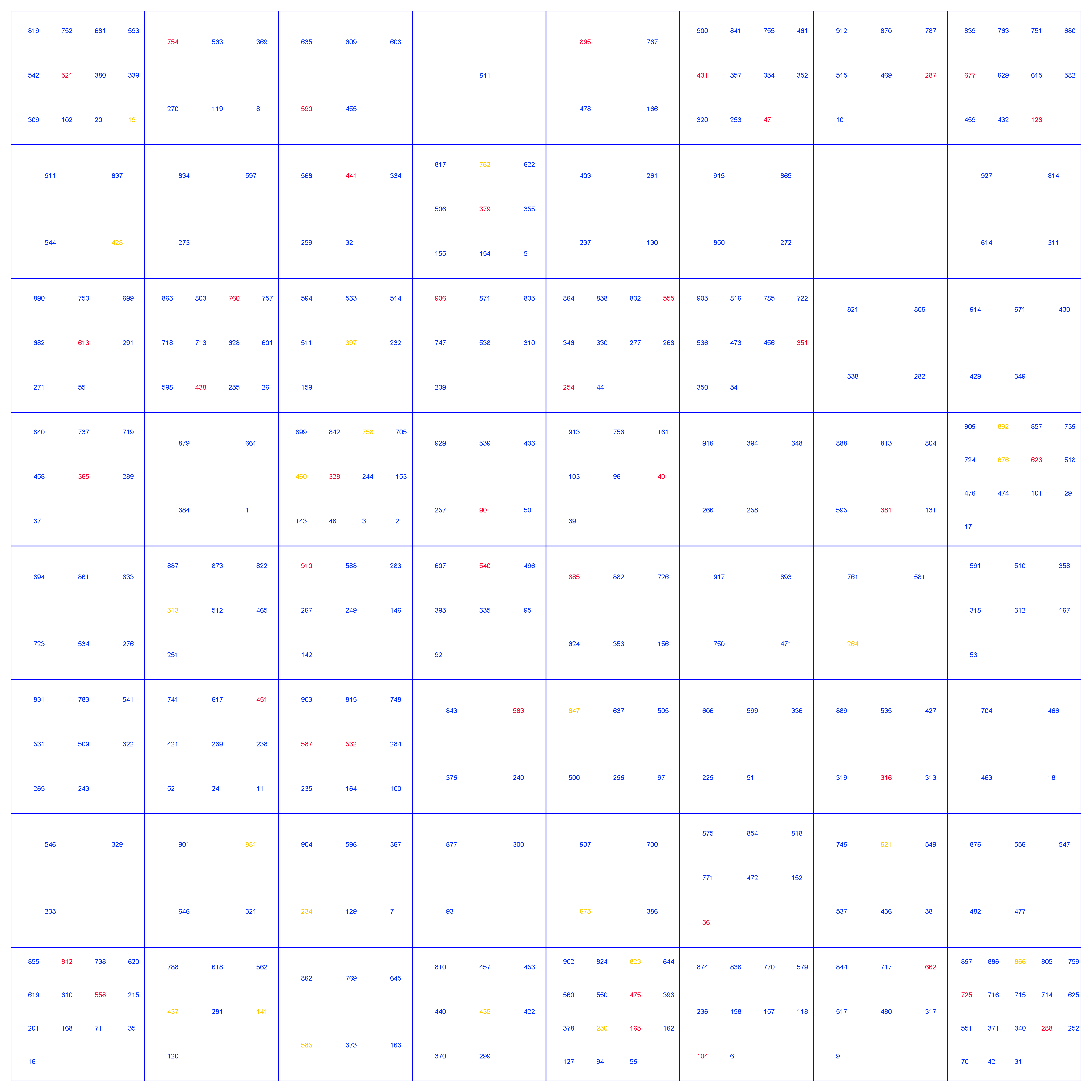

Supplement: Supplementary file 1 [file ijms-22-04443-s001.zip › validation_set_selection.png]
